# New nanocomposites of polystyrene with polyaniline doped with lauryl sulfuric acid

**DOI:** 10.1186/s11671-017-2265-8

**Published:** 2017-08-15

**Authors:** A. A. Pud, O. A. Nikolayeva, L. O. Vretik, Yu. V. Noskov, N. A. Ogurtsov, O. S. Kruglyak, E. A. Fedorenko

**Affiliations:** 1 Institute of Bioorganic Chemistry and Petrochemistry of NAS of Ukraine, 50 Kharkivske shose, Kyiv, 02160 Ukraine; 2 Taras Shevchenko National University of Kyiv, 64/13 Volodymyrska street, Kyiv, 01601 Ukraine

**Keywords:** Polystyrene nanoparticles, Polyaniline, Core-shell nanocomposites, Conductivity, Thermal stability, Sensing ability

## Abstract

This work is concentrated on synthesis and investigation of new core-shell nanocomposites of polystyrene (PS) with doped polyaniline (PANI). The latex containing PS nanoparticles with sizes of 15–30 nm was prepared by microemulsion polymerization of styrene in water media. The PS/PANI nanocomposites were synthesized by chemical oxidative polymerization of aniline in the PS latex media in a presence of lauryl sulfuric acid (LSA), which served as both dopant and plasticizer. The real content of PANI in the synthesized nanocomposites was determined by UV-Vis spectroscopy method. The composition of the nanocomposites and oxidation state of the doped polyaniline were characterized by FTIR spectroscopy. The core-shell morphology of the nanocomposite nanoparticles was proved by transmission and scanning electron microscopy. It was found that conductivity and thermal behavior in air of these nanocomposites not only nonlinearly depended on the doped polyaniline content but also were strongly effected both by plasticizing properties of the acid-dopant and presence of the polyaniline shell. A possibility of application of these nanocomposites as sensor materials has been demonstrated.

## Background

It is well known that polyaniline (PANI) have a unique set of physical and chemical properties, high stability, low price, etc., which allows its multifunctional applications in different high-tech fields such as micro- and optoelectronics, sensor and electrochromic devices, batteries, and supercapacitors, etc. [1, 2]. Processability and applicability of PANI can be significantly improved if it is used in composites or nanocomposites with soluble or meltable common polymers, which can be readily formed into various articles [3]. Among various methods of preparation of such materials, the oxidative polymerization of aniline in water-based acidified latexes or dispersions containing nanoparticles or (sub)micron-sized particles of other polymers (stabilized with different surfactants or non-ionic polymers) is considered as one of the most effective approaches [3]. This approach allows to obtain multifunctional composites or nanocomposites of core-shell type, where the core is the polymer (nano)particle and the shell is formed of PANI [3–10]. To facilitate the formation of the core-shell morphology, aniline is polymerized as its salt, which appears in the latex medium due to aniline interaction with an added acid-dopant, typically HCl (e.g., [8, 10]), or as commercial aniline hydrochloride salt (e.g., [4, 7]). In many cases, stability of the latex polymerization media is additionally supported with non-ionic or ionic stabilizing additives (more often sodium dodecyl sulfate/lauryl sulfate (SDS/SLS) [3–10]). However, there has been developed an alternative method to prepare such materials. This method uses a surface active acid (e.g., dodecylbenzenesulfonic acid––DBSA) unifying properties of surfactants, plasticizers, and acid-dopants and therefore allowing to avoid the abovementioned use of additional HCl or other acid-dopant [11, 12].

Mechanistic aspects of the formation of PANI layers or shells on the surface of different organic or inorganic macroscopic (glass or quartz, polymer films, fibers, etc.) and microscopic (polystyrene latexes, silica or titania, or polymer particles, etc.) substrates (templates) have been discussed in a lot of publications mainly in terms of in situ adsorption polymerization of the positively charged anilinium cations at surface typically bearing negative charges of preadsorbed/grafted anions/functional groups [13–17]. It has been generally accepted that the adsorbed anilinium cations polymerize immediately after the addition of the oxidant-initiator. Naturally, the non-adsorbed anilinium cations are also involved in the polymerization process and form positively charged oligomeric and polymeric molecules, which precipitate/adsorb at the same surface and therefore cause an increase in the PANI shell thickness.

In an alternative approach, aniline monomer was first added to the polystyrene (PS) latex and absorbed in a neutral form by PS core particles for 3 days [9]. After addition of the oxidant-initiator (ammonium persulfate, APS), the conducting polyaniline membrane is formed at the particle interface and separate the both reagents. Electrons are transferred from the aniline molecules to the oxidant molecules through polyaniline membrane, and therefore, polyaniline gradually penetrates inside the PS latex particle, in contrast to the abovementioned core-shell morphology obtained in classical coating of latex particles with polyaniline [9]. In another variation of this approach, after swelling of PS particles with the neutral aniline monomer for 12 h, APS and then hydrochloric acid were added to the reaction medium [18]. The HCl addition resulted in transformation of the aniline molecules released from the particles to anilinium cations, which in turn were polymerized by chemical oxidation with APS. The clear core-shell structure of the formed PS/PANI composite was confirmed in this case [18].

PS quite frequently serves in such materials as the core polymer component due not only to its good thermal and chemical stability, mechanical characteristics, biocompatibility, etc. [19] but probably to its convenience for synthesis of well-shaped nano/submicron/micron-sized particles being very suitable for the (nano)composites with specific applications. For example, micron/submicron-sized PS particles coated with doped PANI were used in electrostatic accelerators, which allowed accelerating the charged particles to hypervelocities [20] or in electrorheological fluids [21], etc. Similar to other core-shell (nano)composites [22] PS/PANI ones obviously have potential for sensing applications. However, to our knowledge, there is a lack of information on using PS/PANI core-shell (nano)composites as sensing materials. Nevertheless, recently, it was shown that mixing PS dissolved in toluene with PANI particles doped with camphorsulfonic acid gave dispersions, suitable for formation of the composite films sensitive to ammonia [23]. Interestingly, while these blends showed quite high responses to gaseous ammonia, i.e., (Δ*R*/*R*_0_) × 100 ~ 73% at 20 ppm, their responses to higher concentrations of ammonia were not very different and were only up to ~ 90% at 100 ppm (Fig. 11 in [23]). This weak response concentration behavior suggests that due to solution preparation of these blends only a part of the sensitive PANI clusters is easily accessible to the analyte molecules, and other part is screened by PS matrix, which imparts some diffusion limitations to the sensing materials. Therefore, one can deduce that PS/PANI core-shell composites with unscreened PANI surface can have an improved sensing behavior as compared with the solution prepared blend materials [23].

Based on the above discussion, our work was concentrated mainly on synthesis of new core-shell nanocomposites of PS nanoparticles with PANI doped with lauryl sulfuric acid (LSA) and on investigation of their practically important properties (morphology, chemical structure, conductivity, thermostability). Their potential applicability as sensing materials was also estimated. The LSA choice is the important feature of the nanocomposites, which is based mainly on three prerequisites: (1) the same lauryl sulfate surface active anion both in the surfactant SLS used at the stage of the core PS nanoparticle synthesis and in the acid-dopant LSA used at the stage of the PANI shell synthesis, (2) it can perform as the surface active functionalized protonic acid-dopant acidifying the reaction medium [24, 25], and (3) it forms anilinium salt (i.e., surface active reactive monomer or surfmer) facilitating formation of nanosized PANI shells and structures [25, 26].

## Methods

### Materials

Aniline (Merck) and styrene (reagent grade, Ukraine) were distilled under vacuum and stored under argon at 3–5 °C. The oxidant potassium persulfate (KPS) (Ukraine), anionic surfactant sodium lauryl sulfate (SLS, synonymously sodium dodecyl sulfate––SDS, Aldrich) were of reagent grade and used without further purification. Lauryl sulfuric acid (LSA) was prepared from the SLS via ion-exchange reaction with KU-2-8 resin (Ukraine).

### Preparation of PS latexes

PS nanoparticulate latexes were prepared by radical polymerization of styrene in accord with the method described elsewhere [27]. In short, styrene was polymerized in a micellar aqueous solution of SLS with oxidant-initiator KPS as follows: 2 g of styrene were added slowly over a period of 1.5 h to a vigorously stirred solution of 0.01 g NaH_2_PO_4_, 0.2 g SLS, and 0.01 g KPS in 10 ml of water at 70 °C in argon atmosphere. The mixture was stirred for additional 3 h at 70 °C and then for additional 1 h at 90 °C. The final polymerization mixture was cooled to room temperature and purified by dialysis through cellulose membrane with MWCO 3500 Da against distilled water for 48 h.

### Preparation of PS/PANI-LSA nanoparticles

The aniline polymerization in the PS latex was carried out similarly to the method described elsewhere [28, 29] at next ratios of the reaction mixture components: aniline/LSA = 1/1.5 (mol/mol) and aniline/oxidant = 1/1.25 (mol/mol) at 10 °С. The initial weight ratio of aniline to PS nanoparticles in the polymerization mixture was predetermined by expected theoretical quantities of dedoped polyaniline in the ultimate nanocomposites in a range of 1–10 wt%. In short, at the first stage of the preparation of the polymerization mixture, a calculated quantity of the acid was added to the target PS latex portion and stirred at room temperature for 30 min. At the second stage, the calculated quantity of aniline was added to this acidified PS latex followed by stirring for 1 h to allow complete formation of the anilinium salt at room temperature and then the prepared mixture was cooled down to 10 °C for 30 min. At the third stage, the calculated quantity of the precooled to 10 °C KPS solution in distilled water was added dropwise into the reaction mixture followed by stirring for 24 h at 10 °C. After the aniline polymerization was completed, the obtained PS/PANI-LSA latexes were purified by dialysis through the cellophane membrane against distilled water for 3 days. The purified nanocomposites were dried at ambient conditions to visually dry powder condition followed by drying under vacuum at 60 °C until a constant weight was reached. The reference pure PANI-LSA sample was synthesized under the same conditions in the water solution in the absence of PS nanoparticles.

### Characterization

The real PANI contents in the synthesized nanocomposites were determined similarly to [29] by UV-Vis spectroscopy analysis of their solutions in in N-methyl-2-pyrrolidone (NMP) with the help of spectrophotometer Cary 50 (Varian). In short, at the first stage, the dry nanocomposite was typically dedoped in 0.3 wt% ammonia aqueous solution for 24 h followed by washing with distilled water and then drying under vacuum at 60 °C until a constant weight was reached. At the second stage, the fixed portion of the dedoped powder nanocomposite was dissolved in NMP and mixed with ascorbic acid solution in NMP to obtain leucoemeraldine base (LB, fully reduced form of PANI). At the third stage, the LB concentration was calculated from UV absorption of this solution in 1 mm quartz cuvette at 343 nm using the previously prepared calibration curve. At the fourth stage, this LB concentration was then recalculated for the real dedoped PANI content in the nanocomposite. The latter content was then used to estimate the doped PANI-LSA content in this PS/PANI-LSA nanocomposite. This final recalculation was based on the theoretical stoichiometric ratio of LSA and imine nitrogens in PANI namely PANI:LSA = 1:0.5 similarly to [28, 29]. In accord with [28], for simplicity and clarity of this recalculation, we postulated the complete PANI doping with only LSA. The compositions of the synthesized nanocomposites and their notations are given in Table 1.

**Table 1:** Description of the samples

Samples	Real PANI base content, wt%	PANI-LSA content, wt%	Notation^a^
PANI-LSA	Reference sample		
PS/PANI-LSA	0.75	1.84	NC2
PS/PANI-LSA	1.24	3.01	NC3
PS/PANI-LSA	2.45	5.84	NC6
PS/PANI-LSA	4.89	11.27	NC11
PS/PANI-LSA	6.58	14.82	NC15

^a^NC is a general abbreviation of the nanocomposite; numerals display the rounded calculated content of PANI-LSA based on the real dedoped PANI contents

Fourier transform infrared (FTIR) spectra of the PS/PANI-LSA nanocomposites and pure PANI-LSA samples in pellets with KBr were recorded with a resolution of 1 cm^−1^ with Bruker Vertex 70 spectrometer.

Transmission and scanning electron microscopy (TEM and SEM) images were obtained with JEOL JEM-1400 and Hitachi S4800 microscopes, respectively. Samples for TEM measurements were prepared by placing 2 μL of the sample water dispersion onto a carbon or formvar coated 200 mesh copper grids for 15 min followed by a careful removal of the dispersion with a filter paper. The samples for SEM measurements were prepared by dropping 5 μl of the dialyzed water dispersion of pure PS or a nanocomposite onto a glass plate. The dried samples were sputter coated with a thin (~ 7 nm) gold layer.

Thermal stability of the synthesized materials was studied by thermogravimetry analysis (TGA) of their samples in air when using a MOM Q-1500 D (Paulik-Paulik-Erdey) Derivatograph system with a heating rate of 10 °C/min.

In order to characterize conductivity properties of the synthesized nanocomposites, their powders were processed into films both by compression molding technique at 240 °C under 5 MPa (using SPECAC press) for 2 min and by casting on glass plates from their 3% dispersions prepared under ultrasonication.

To estimate applicability of the synthesized PS/PANI-LSA nanocomposites as materials which are sensitive to harmful gases, we used the most conducting nanocomposite NC15 and compared its properties with pure PANI-LSA synthesized under the same conditions. Ammonia-air mixtures with ammonia concentrations in the range of 19–152 ppm served as analytes. Sensitive elements were prepared as follows. A 1 μL volume of the ultrasonically treated dispersions of the nanocomposites in solvent (2% *w*/*v*) was drop-cast on the miniature system of gold interdigitated electrodes formed on the glass–ceramic substrate. The formed sensing elements were dried at 60 °C for 30 min and then installed into the airtight testing chamber described elsewhere [30]. The prepared ammonia-air mixtures were injected by syringe in this chamber. Sensor responses (SR) of these elements were recorded at ambient temperature and relative humidity around 50% and determined as a relative variation of the resistance *R* of the sensor exposed to the analyte in accord with the equation SR = [(*R*−*R*_0_)/*R*_0_] × 100%, where *R* is the sample resistance, *R*_0_ is the initial resistance value.

## Results and discussion

### Morphology of the synthesized PS/PANI-LSA nanocomposites

**Fig. 1:**
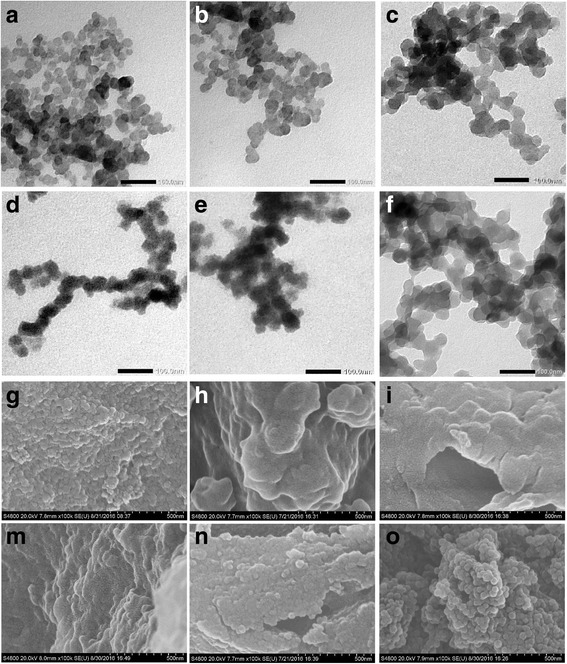
TEM (**a**–**f**) and SEM (**g**–**o**) images of pure PS and PS/PANI-LSA nanocomposites: **a**, **g**- pure PS; **b**, **h**- NC2; **c**, **i**- NC3; **d**, **m**- NC6; **e**, **n**- NC11 and **f**, **o**- NC15

As one can see from the TEM image (Fig. 1a), the used synthetic approach allowed to synthesize spherically shaped PS nanoparticles of very small sizes in the range of 15–30 nm. To our best knowledge, these PS nanoparticles are among the lowest PS ones.

TEM images of the PS/PANI-LSA nanoparticles separated after the polymerization show that they have sizes increasing with PANI-LSA content (Fig. 1b–f). This effect suggests core-shell morphology of these nanoparticles with core of PS nanoparticle and shell of PANI-LSA. Nevertheless, despite the increased sizes, in the case of low contents of PANI-LSA in the nanocomposites (NC2, NC3, NC6), it is quite difficult to visually distinguish thin PANI-LSA shells (Fig. 1b–d). This problem can be probably explained by a polymer nature of the both components and loose structure of these shells. The latter, in turn, can be caused by the large size of the dopant anions hindering the formation of compact PANI-LSA shells. However, at higher PANI-LSA contents in NC11 and especially in NC15 the irregular shells can be distinguished (Fig. 1e, f).

In spite of quite wide size distributions of the nanocomposite particles (Fig. 1b–f), we can roughly estimate their shell thicknesses. In particular, while NC2 with the lowest content of PANI-LSA (Table 1) contains nanoparticles with sizes around 15 nm similar to those of parent PS, one can find in the TEM image (Fig. 1b) nanoparticles with sizes up to 40 nm that probably indicates presence of PANI-LSA shells with thicknesses up to 10 nm on their surfaces.

In the case of NC3 15 nm nanoparticles are not observed but the number of 30–40 nm nanoparticles with shell thicknesses up to 10 nm increased significantly (Fig. 1c). This tendency is enhanced in NC6 nanoparticles (Fig. 1d). TEM images of NC11 and especially of NC15 display nanoparticles with increased sizes in the range of about 25–50 nm (Fig. 1e, f). A presence of some spots of irregular shape suggests appearance of a separate phase of PANI-LSA in these nanocomposites due to its higher contents. Moreover, the NC15 image allows to clearly distinguish irregular PANI shells with thicknesses of 10–20 nm.

After cleaning and preparation of the parent PS latex for SEM imaging (see “Characterization” section), PS nanoparticles formed agglomerates with sizes in the range of 30–150 nm or more, which presumably included 2–5 or more initial nanoparticles (Fig. 1g). The aniline polymerization in the latex medium in the presence of surface active LSA changed the situation (Fig. 1 h–o). Thus, at the lowest PANI-LSA content (1.84 wt%), one can see in the NC2 image large irregular entities with sizes of about 400–500 nm which have a quite smooth surface (Fig. 1h). In the case of NC3 with increased PANI-LSA content (3.01 wt%), the entities have a tendency to less sizes in the range of about 100–300 nm. This tendency is strongly enhanced at higher PANI-LSA content in NC6 (5.85 wt%). In particular, its SEM image shows not only a small number of entities with sizes up to 150 nm but also irregular agglomerates with sizes in the range of 40–100 nm (Fig. 1m). SEM images of NC11 and NC15 (Fig. 1n, o) demonstrate further development of the samples morphology, namely, qualitative and quantitative changes in these nanocomposites due to the highest PANI-LSA contents 11.27 and 14.82 wt%, respectively. Specifically, one can see quite densely packed agglomerates with sizes mainly in the range of about 25–50 nm on the flat NC11 sample surface, while in the case of the NC15 sample well distinguishable 25–50 nm agglomerates arranged in “bunch of grapes” morphology are observed (Fig. 1n, o). This morphology suggests a higher specific surface of NC15 compared with other nanocomposites.

In general, the TEM and SEM measurements show that although pure PS nanoparticles after cleaning tend to agglomerate, the low contents of PANI-LSA in the nanocomposites NC2 and NC3 suppress this agglomeration. This effect can be assigned to the surface activity of charge compensating large LS¯ anions which localize around positively charged PANI shells on PS cores and therefore separate the nanoparticles. However, the situation is reversed at the moderate (NC6) and especially at the high (NC11 and NC15) PANI-LSA contents, which apparently facilitate formation of quite thick PANI-LSA shells around PS cores. As a result, number of charge compensating LS^¯^ anions both around and inside of positively charged PANI shells becomes higher as compared with NC2 and NC3 cases. Inevitably, these amphiphylic anions with long dodecyl tails can enhance existing in the system intermolecular interactions. These interactions are probably stronger that the abovementioned tendency in NC2 and NC3 and in turn can cause the observed agglomeration of NC6, NC11, and NC15 nanoparticles.

### FTIR measurements

**Fig. 2:**
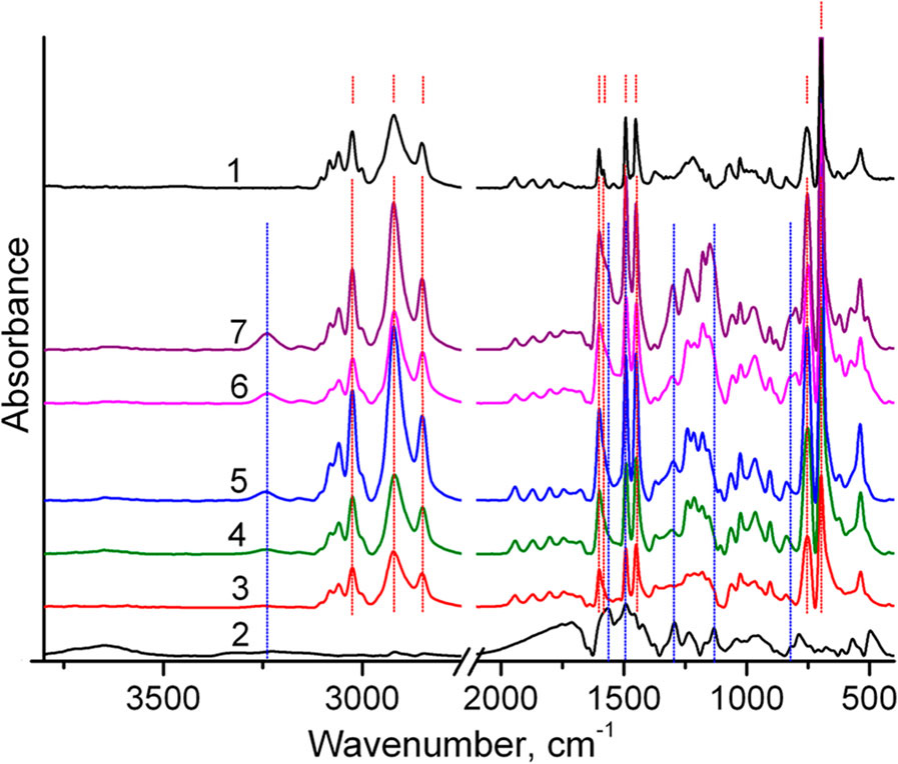
FTIR spectra of PS (1), PANI (2), and PS/PANI-LSA composites: NC3 (3), NC3 (4), NC3.5 (5), NC11 (6), NC15 (7). Main characteristic peaks of PS and PANI-LSA are marked with *dashed red* and *blue lines*, respectively. All marks correspond to the frequencies discussed in the text

The structures of the synthesized polymers are characterized by means of their FTIR spectra. In particular, as one can see in Fig. 2, FTIR spectrum of PS contains five characteristic peaks of aromatic C–H stretching vibrations with the maximal peak at 3025 cm^−1^ [31]. Peaks of C–H stretching vibration of methylene groups occur at 2920 and 2850 cm^−1^. Four bands of aromatic C=C stretching vibrations are observed at 1601, 1583, 1492, and 1452 cm^−1^. The very strong bands at 756 and 697 cm^−1^ can be assigned to the CH out-of-plane vibration and the ring out-of-plane deformation, respectively, [31]. These bands confirm the presence of a monosubstituted aromatic group.

In turn, FTIR spectrum of PANI-LSA agrees well with published data [32–34]. It contains typical bands at 1565, 1492, 1294, 1133, and 818 cm^−1^ assigned to stretching vibrations of quinoid rings, benzenoid rings, C–N stretch in a secondary aromatic amine, vibrational mode of a B–NH^+^ = *Q* structure, C–H out-of-plane bending of 1.4-rings, respectively. Some features, such as a very weak NH stretching vibrations in the region 3l00–3500 cm^−1^, indicate that PANI is in the doped state. However, the B–NH^+^ = *Q* band intensity at 1133 cm^−1^ is quite weak that suggests a quite low doping level of this PANI-LSA [34].

**Fig. 3:**
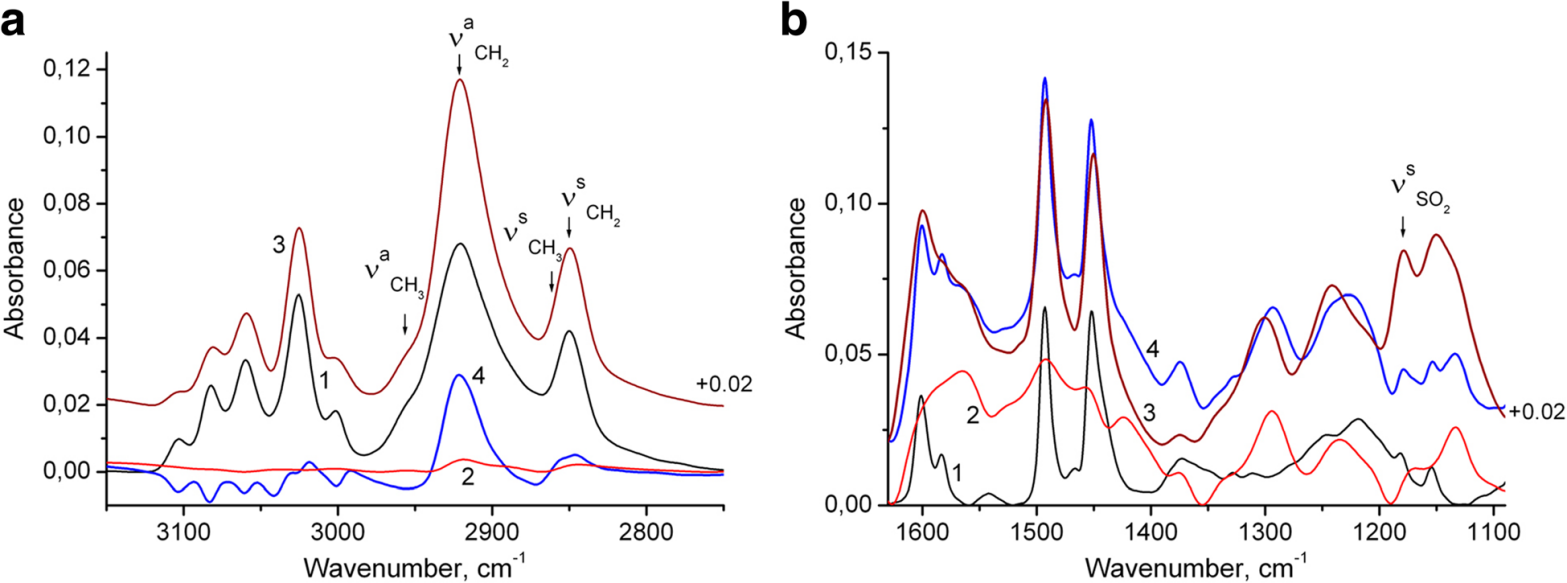
FTIR spectra of PS nanoparticles (1), PANI-LSA (2), and NC15 (3): **a** spectrum 4 is the result of subtraction of the normalized PS spectrum from the NC15 one, **b** spectrum 4 is the sum of the PS spectrum (normalized to the band height of NC15 at 3025 cm^−1^), and the PANI-LSA spectrum (normalized to the band height of NC15 at 1560 cm^−1^)

A distinct band at about 1180 cm^−1^ (Figs. 2 and 3b, curve 1) originating from S=O stretching vibration [31] shows that synthesized PS nanoparticles contain lauryl sulfate anions, which are present apparently due to synthesis conditions of the PS nanoparticles (see Methods part). Moreover, an excess of these anions is evidently observed in the final PS/PANI-LSA composites. Thus, as can be seen from Fig. 3a (curve 2), C–H stretching vibrations of aromatic rings and methylene groups of PANI-LSA are very weak. Therefore, the intense bands of C–H stretching vibrations of methylene groups, which are revealed due to subtraction of the PS spectrum from the NC15 one (after normalization by height of the band at 3025 cm^−1^) (Fig. 3a, curve 4), can be obviously assigned to the separate SLS phase.

To evaluate the state of PANI in the nanocomposite, we compared the spectrum of NC15 with the model spectrum (Fig. 3b, curves 3 and 4 accordingly). The last is the sum of the PS and PANI-LSA spectral contributions. In particular, the PS contribution is the PS spectrum normalized to the band height of NC15 at 3025 cm^−1^ (where the PANI-LSA absorption is very weak), and PANI contribution is the PANI spectrum normalized to the band height of NC15 at 1560 cm^−1^ (where the PS absorption is absent). It is known that doped PANI bands at about 1580 and 1490 cm^−1^ have a major contribution from the quinoid and benzenoid rings, respectively, [32–34]. The intensity ratio of these bands is sensitive to the chemical structure of the PANI, and therefore, the dominance of quinoid rings over the benzenoid units in the spectrum of NC15 compared with the model spectrum testifies that the oxidation degree of PANI-LSA phase in the nanocomposite is higher than that of the pure PANI. One can also see that the PS bands of the aromatic C=C stretching vibrations at 1601 and 1583 cm^−1^ are broadened in the NC15 spectrum and slightly shifted to lower wavelengths. This shift probably indicates π–π interaction between PANI and PS. The intensity of the NC15 band at 1133 cm^−1^ is appreciably higher than that of the model spectrum, indicating the higher conductivity of PANI phase in this nanocomposite as compared with the pure PANI.

### Thermal stability

**Fig. 4:**
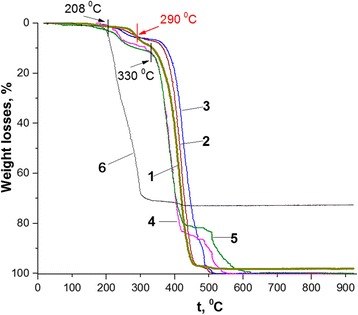
Thermogravimetric curves of the PS/PANI-LSA nanocomposites with different PANI-LSA contents (wt%): **1** PS, **2** 1.84 (NC2), **3** 3.01 (NC3), **4** 11.27 (NC11), **5** 14.82 (NC15), **6** SLS

Recently, it has been shown for micron-sized particulate core-shell polymer-polymer composites of polycarbonate (PC) with quite low contents of PANI (~ 2 wt% of PANI base or 3.5–5.0 wt% if doped by different aromatic sulfonic acid-dopants) that presence of the dopant strongly affects their thermal stability [35]. Depending on the alkyl substituent in aromatic ring of the dopants, the composites demonstrated more (long substituent) or less (short substituent) decrease of their thermal stability as compared with pure PC due to specific intermolecular interactions of the plasticizing large dopant anions and/or thermally released dopant molecules with PC chains [35]. But at temperature above 500 °C, at which PANI in the shell is typically in the dedoped (base) state, the composites stability was higher than that of PC. This effect was assigned to a specific state of PANI located as a shell at the surface of the core material in the core-shell composites and to a possible stabilization of the PC core particle by the PANI shell [35, 36]. Based on this possibility and the nanocomposites morphology, we suggest that the PANI shell stabilizing effect can also take place for different polymer-PANI core-shell nanocomposites. The suggestion agrees well with thermal behavior of the synthesized PS nanoparticles and nanocomposites illustrated in Fig. 4.

Indeed, the synthesized PS nanoparticles demonstrate thermal stability differing in some extent from that of the bulk PS (compare curve 1 in Fig. 4 and Figure 1 in [37]). In particular, while the latter degrades in air primarily in a single step from 200 to 450 °C [37], the thermogravimetric (TG) curve of the former shows roughly three stages: week weight losses (~ 1.9 wt%) from beginning to 262 °C, the second one in the range of 262–330 °C and the third one in the range of 330–505 °C. This difference can be probably explained by specificity of PS nanoparticle synthesis resulted in inevitable presence in their composition of SLS impurity which in turn changed the PS thermal behavior. This suggestion agrees well with the fact that the final degradation temperature of SLS is very close to the beginning (330 °C) of the third (main) stage of the PS nanoparticles degradation (Fig. 4, curves 1 and 6).

As one can see in Fig. 4, TG curves of the nanocomposites have a shape similarity with that of PS and, moreover, demonstrate similar small mass losses at temperatures up to 120 °C, which typically can be assigned to water evaporation [35]. At higher temperatures, one can see significant differences in thermal stability of the samples with low and high loading of PANI-LSA, which, in general, give complementary information about the thermal behavior specificity of the known core-shell PANI-containing composites. In particular, three of the nanocomposites (NC2, NC3, and NC11) with PANI-LSA content ≤  11.27 wt% display similar to PS high thermal stability up to 208 °C (Fig. 4, Table 2). However, NC15 with the highest PANI-LSA content (14.82 wt%) is less stable than PS even at 120 °C (Fig. 4, curves 1 and 5; Table 2) that probably can be assigned also to evaporation of not only moisture but also probably to the unbound dopant and/or unreact monomer/oligomer impurities [38].

**Table 2:** Weight losses (in wt%) of the PS/PANI-LSA nanocomposites (see Table 1), control PS nanoparticles, and SLS samples at different temperatures. LSA contents are given in brackets

Temperature, °C	NC2 (1.09)	NC3 (1.77)	NC11 (6.38)	NC15 (8.24)	PS	SLS
120	0.12	0.12	0.18	0.9	~0	0.61
180	0.96	0.64	0.85	2.12	0.8	1.29
208	1.33	1.33	1.33	2.93	1.33	3.92
262	3.45	4.59	7.64	8.78	1.93	39.98
290	5.68	5.68	8.74	10.27	5.68	58.06
330	7.04	6.65	12.01	12.01	9.33	70.50
350	7.83	7.06	15.86	15.86	12.80	71.00
430	69.42	50.68	83.56	80.50	82.04	72.86
505	99.42	98.65	91.01	83.36	97.5	73.04

In the temperature range of 208–262 °C, all nanocomposites show weight losses, which are higher than LSA contents but significantly less than weight losses of PS (Fig. 4, curves 1, 4, and 5, Table 2). However, in the case of NC2 and NC3, these losses are even higher than contents of PANI-LSA. Based on the high thermal stability of PANI base [39] and thermal behavior of the PS nanoparticles (Fig. 4, curve 1), we probably may assign the nanocomposite losses not only to evaporation and degradation of the dopant but also to degradation of the PS component. Moreover, whereas weight losses of NC2 and NC3 at 262 °C (Table 2) exceed sums of their LSA contents and PS loss (3.02 and 3.7, respectively), one may assume that some enhancement of the thermooxidative degradation of the PS core component of the nanocomposites can be caused by degradation products of the dopant.

Although the nanocomposites losses typically increase at higher temperatures, at 290 °C TG curves of NC2 and NC3 (unlike those of NC11 and NC15) intersect with TG curve of PS in the point of 5.58 wt% (Fig. 4, Table. 2). This behavior, in general, suggests a complete loss of the dopant [35, 37, 38] and transformation of the PANI-LSA component in the dedoped PANI. Above this, temperature NC2 and NC3 are more stable than PS up to the end of the heating process (Fig. 4, curves 1–3). As a consequence, the position of the TG trace of PS nanoparticles along the temperature axis in the range of 262–430 °C roughly separates positions of the nanocomposites with low and high contents of PANI-LSA (Fig. 4). This fact confirms a difference which is probably inherent to these two sets of nanocomposites.

Indeed, one can see strongly different course of the thermal degradation of these nanocomposites both in the range of 262–430 °C and above 430 °C. Whereas all these nanocomposites have the core-shell morphology, it is unlikely that only this morphological factor can explain their specific thermal behavior. However, if to take into account the presence in their composition of the LSA dopant, which contains the long dodecyl tail with plasticizing ability [3], we can at least partially understand such difference as a result of intermolecular interactions (causing a plasticizing effect [40]) of the dopant anion with the polymer components of the nanocomposites. Naturally, in the case of low or high contents of the PANI-LSA component, its influence on thermal behavior of the nanocomposites will be less (NC2, NC3) or more (NC11, NC15) significant. In the latter case, the plasticization effect is so strong that the thermogramms of NC11 and NC15 (Fig. 4, TG curves 4 and 5) take positions below the PS one up to 430 °C even after a complete removal of the dopant (above ~ 290 °C) because of weakened interactions between PS macromolecules. Slowing down the degradation rate of the nanocomposites with high content of PANI base at temperatures above 430 °C can be probably explained by cross-linking of its chains [39] and possible enhancement of the stabilizing role of the PANI shell.

In the case of NC2 and NC3, the situation is obviously opposite to NC11 and NC15. In particular, contents of PANI-LSA are quite small, and therefore, quantities of the plasticizing dopant LSA are not enough to significantly weaken interactions between PS macromolecules. As a consequence, once the dopant is eliminated completely, the nanocomposites display thermostability which is higher than that of PS nanoparticles (Fig. 4, curves 2 and 3, intersection point at 290 °C). In spite of the low content of PANI-LSA and, therefore, of its thin shell, these NC2 and NC3 behaviors match well with the suggestion about stabilizing effect of the PANI base shell.

### Conductivity and sensing properties of the synthesized nanocomposites

**Fig. 5:**
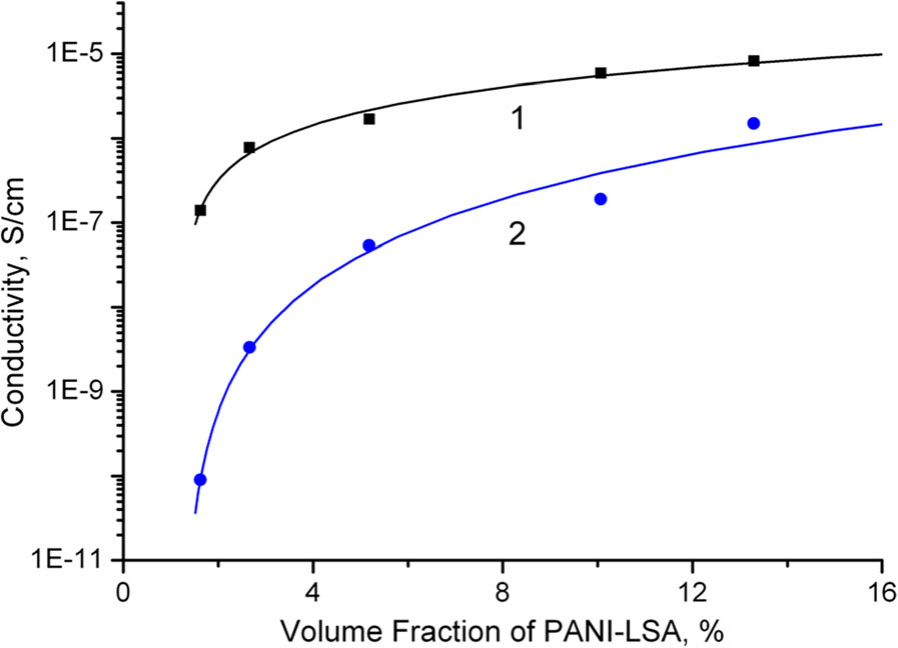
Dependencies of DC conductivity of the cast (**1**) and compression molded (**2**) PS/PANI-LSA nanocomposite films on the volume fraction of PANI-LSA

One of most important features of polymer-polymer composites, in particular of PANI-containing composites, is probably their ability to withstand conditions of common treatments, which are typically applied to produce different articles. Therefore, a lot of studies have been performed to estimate changes in properties of these materials after treatments by melting or solution techniques [3, 38, 39]. Based on these studies and the thermally induced weight losses of the synthesized nanocomposites (Table 2, Fig. 4), one might expect that such important property of doped PANI as conductivity could be changed under these treatments. Indeed, as one can see from Fig. 5, the values of conductivity of the cast and compression molded PS/PANI-LSA films strongly differ. To quantify the difference, we treated these data (Fig. 5) by the scaling law based on the percolation theory [41] in accordance with the known methodology of processing the conductivity behavior of polyaniline networks in composites [42, 43]:

1 $$ \sigma ={\sigma}_{\mathrm{o}}{\left(f-{f}_c\right)}^t $$

where *σ*_o_ is the constant displaying conductivity of the PANI conducting phase, *f* is the volume fraction of PANI, *f*_*c*_ is the percolation threshold, and *t* is the critical exponent. Volume fractions of PANI-LSA in the nanocomposites were calculated on the basis of densities of PS and PANI-LSA, i.e., 1.04 [44] and 1.18 g/cm^3^ [45], respectively.

The power-law dependence was determined with various trial values of *f*_*c*_ by applying a linear regression analysis to the plot of log *σ* versus log (*f* − *f*_*c*_). The solid lines represent best fits to the data with the correlation coefficients of 0.996 and 0.993 for the cast and compression molded nanocomposite films, respectively.

The observed nonlinear dependences (Fig. 5) are obviously the result of formation of the phase-separated conducting percolation network of PANI-LSA in the bulk of the nanocomposite films. It is interesting to note that the percolation thresholds are quite low (*f*_*c*_ = 1.26%) and independent on the used processing techniques. This *f*_*c*_ value is significantly lower than the theoretical model suggests for a random lattice of spheres (from 15 to 30% depending on the sphere diameter) [41]. However, the conductivity of the PANI conducting phase (*σ*_o_) in the cast nanocomposite films is more than two and a half times higher than that of the compression molded ones (2.3 × 10^−4^ and 8.9 × 10^−5^ S/cm, respectively). Obviously, the lower conductivity of the conducting phase in the compression molded film is caused by the partial thermal degradation of PANI-LSA under the melting treatment temperature (240 °C). The values of the critical exponent *t* for the cast and compression molded films are 1.14 and 2.62 accordingly. Such inequality in the critical exponent indicates a strong difference in the spatial structure of the percolation cluster, which results in the different slopes of the curves. As a consequence, the conductivities of the cast nanocomposite films are more than three orders of magnitude higher than those of the compression molded ones at low volume fractions (contents) of PANI-LSA (Fig. 5).

Nevertheless, despite the significant difference of the cast and compression molded films, one can deduce that the conductivity level is enough to apply the both materials for antistatic applications. On the other hand, the obtained conductivity values of the synthesized nanocomposites are significantly lower (by 2–3 orders of magnitude) than in the case of the similar core-shell submicron/micron-sized PS/PANI composites [4, 7, 9, 14]. To understand this difference and to improve the conductivity of these new PS/PANI-LSA nanocomposites, new studies are planned. However, one can suggest that non-optimal conditions of preparation of these new materials are at least partial explanation of this low conductivity level.

Based on better conductivity properties of the cast PS/PANI-LSA films and known high sensing ability of doped PANI [46], we estimated their potential as sensing materials to determine concentrations of ammonia in its gaseous mixtures with air. The measurements were performed on the example of the films of NC15 and pure PANI-LSA cast on electrodes of the transducer (see “Methods” chapter).

**Fig. 6:**
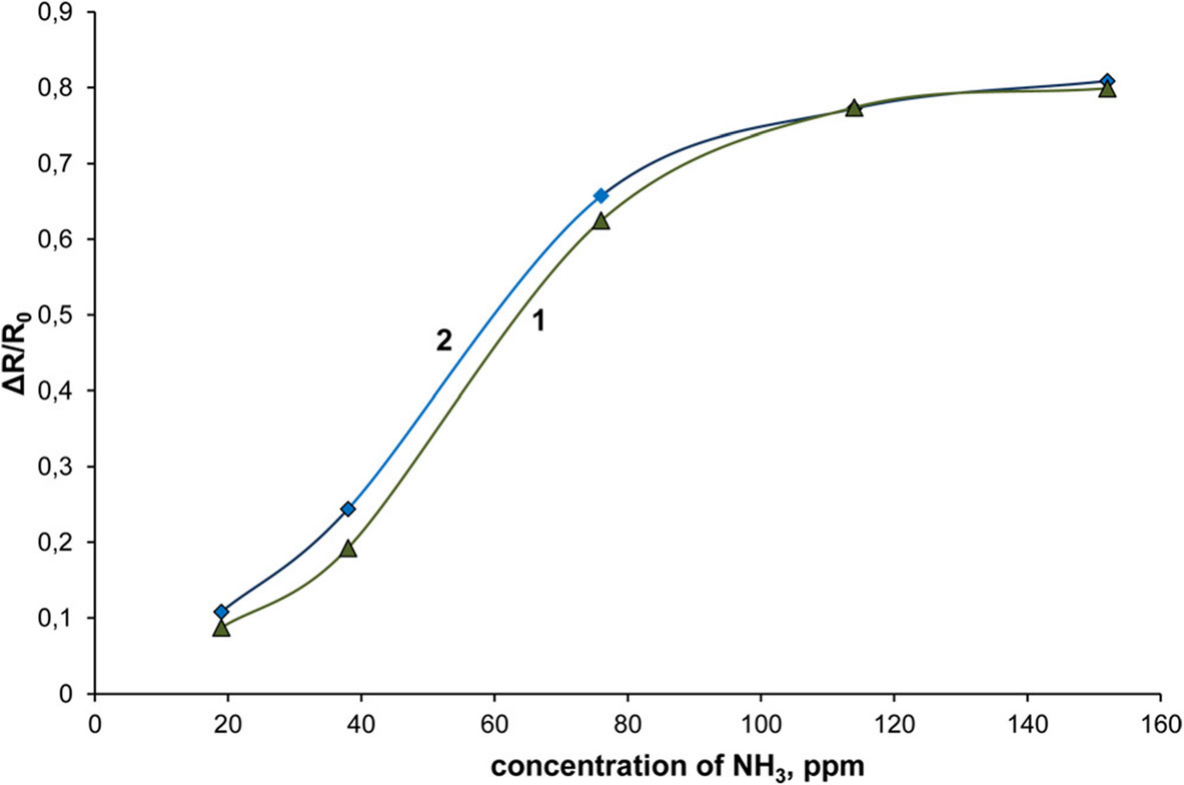
Sensor responses (calibration curves) of the cast pure PANI-LSA (**1**) and NC15 (**2**) films to different concentrations of ammonia in the mixtures with air

Both films demonstrate quite high sensitivity to ammonia in the range of 19–152 ppm (Fig. 6). However, while NC15 is more sensitive to ammonia than pure PANI-LSA in the concentration range of 19–114 ppm, at higher concentrations, the situation becomes opposite.

The better efficiency of NC15 in this narrowed ammonia concentration range can be probably assigned to core-shell morphology of the nanoparticles constituting the cast nanocomposite film. This morphology typically specifies higher surface of the film as compared with pure PANI-LSA and improves sensitivity of sensing materials [25, 28]. The enhancement of the sensing responses of pure PANI-LSA at ammonia concentrations above 114 ppm (Fig. 6, curve 2) can be probably assigned to additional involving in the sensing process of the PANI-LSA clusters located under the surface of the film. Naturally, the quantity of these clusters in the pure doped PANI film is much higher than in the case of the thin PANI-LSA shells on the core particles constituting the nanocomposite film. Therefore, their involvement in the sensing process inevitably increases sensor responses of the pure doped PANI film as compared with the NC15 one.

## Conclusions

The new PS/PANI-LSA nanocomposites have been synthesized with the core-shell nanoparticle sizes ~ 25–50 nm, which to our knowledge are the lowest ones among the similar composites published elsewhere. The use of LSA as acidifying agent for the aniline containing PS latex medium and addition of the oxidant resulted in the precipitation of the thin PANI-LSA shell (~ 10–20 nm) on the surface of the PS nanoparticles (synthesized in the presence of SLS). As a consequence, both the shell and PS core contained the same lauryl sulfate surface active anion unlike the known core-shell PS/PANI composites synthesized with a PS latex surfactant-stabilizer and PANI dopant of different nature.

We have found that although the synthesized very small PS nanoparticles (15–30 nm) after cleaning tend to agglomerate in the dry state, the low contents of PANI-LSA in the nanocomposites suppress this agglomeration probably due to the surface activity of charge compensating large LS¯ anions which localize around positively charged PANI shells on PS cores and separate the nanoparticles. However, the situation becomes opposite at the moderate and especially at the high PANI-LSA contents, which apparently facilitate formation of quite thick PANI-LSA shells around PS cores. In this case, a number of charge compensating LS^¯^ anions both around and inside of the positively charged PANI shells is higher as compared with the low contents of PANI-LSA in the nanocomposites. As a consequence, these amphiphylic anions with long dodecyl tails can enhance intermolecular interactions in the system and lead to the agglomeration of the nanoparticles with high contents of PANI-LSA.

A possibility of such agglomeration effects should be taken into account when using similar nanocomposites in applications which need charged nanoparticles [7, 20, 25]. We believe that applied in this work method of determination of the real PANI content in the PS/PANI nanocomposites can allow better control of their properties.

Based on FTIR and conductivity studies of the synthesized nanocomposites, we proved that oxidation state and conductivity of the PANI phase are appreciably higher than those of pure PANI-LSA. Moreover, we demonstrate here that thermal behavior of these nanocomposites in air is strongly different for low and high PANI-LSA loadings that probably stems both from the plasticizing ability of the LSA dopant and stabilizing effect of the PANI shell. This fact, in general, gives the complementary information about the thermal behavior specificity of the known PANI containing core-shell composites.

At the same time, based on thermal stability, conductivity and sensor studies, we conclude that properties of the synthesized PS/PANI-LSA nanocomposites testify to their potential applicability as materials for antistatic and sensing applications.
